# Comprehensive evaluation of the MeltPro MTB/PZA assay for prediction of pyrazinamide resistance in multidrug-resistant tuberculosis

**DOI:** 10.1128/spectrum.02745-24

**Published:** 2025-05-22

**Authors:** Guiqing He, Chunxia Tu, Yelei Zhu, Qingyong Zheng, Qiulong Zhou, Wenzhen Zhou, Ouyang Huang, Bin Chen, Zhengwei Liu, Ye Xu, Xiangao Jiang

**Affiliations:** 1 Department of Infectious Diseases, Wenzhou Central Hospital, The Dingli Clinical College of Wenzhou Medical University, Wenzhou, China; 2 Laboratory of Infectious Diseases, Wenzhou Central Hospital, The Dingli Clinical College of Wenzhou Medical University, Wenzhou, China; 3 Engineering Research Centre of Molecular Diagnostics of the Ministry of Education, State Key Laboratory of Cellular Stress Biology, School of Life Sciences, Faculty of Medicine and Life Sciences, Xiamen University 199198, Xiamen, Fujian, China; 4 Engineering Research Centre of Molecular Diagnostics of the Ministry of Education, State Key Laboratory of Molecular Vaccinology and Molecular Diagnostics, School of Life Sciences, Faculty of Medicine and Life Sciences, Xiamen University 199198, Xiamen, Fujian, China; 5 Department of Tuberculosis Control and Prevention, Zhejiang Provincial Center for Disease Control and Prevention, Hangzhou, China; Weill Cornell Medicine, New York, New York, USA

**Keywords:** multidrug-resistant TB, pyrazinamide, *pncA*, MeltPro MTB/PZA assay, pDST

## Abstract

Resistance to pyrazinamide (PZA) poses significant challenges to tuberculosis (TB) management, and prediction of susceptibility to PZA has been challenging. This study examined PZA resistance-associated gene mutations in 125 multidrug-resistant clinical isolates of *Mycobacterium tuberculosis* (MTB) from Wenzhou, China. Phenotypic drug-susceptibility testing (pDST) for PZA was performed on clinical isolates using the MGIT 960 system to accurately determine resistance. Subsequently, whole-genome sequencing (WGS) was conducted on the 125 isolates to identify genetic mutations linked to PZA resistance, focusing specifically on the *pncA*, *rpsA*, and *panD* genes. To provide a rapid diagnostic alternative to traditional methods, the MeltPro MTB/PZA assay was also utilized to detect mutations in *pncA*. pDST revealed a PZA resistance rate of 59.20%, with 29.41% observed in strains resistant only to isoniazid and rifampicin, 77.61% in pre-extensively drug-resistant TB (pre-XDR-TB), and 100% in extensively drug-resistant TB (XDR-TB). Among the isolates, WGS identified mutations primarily in the *pncA* (64.00%) and *rpsA* (6.40%), with *panD* mutations not detected. PZA resistance was strongly associated with *pncA* mutations, present in 97.30% of PZA-resistant strains. WGS demonstrated 97.30% sensitivity and 84.31% specificity compared to pDST, while MeltPro MTB/PZA showed 91.89% sensitivity and 86.27% specificity. Compared to WGS, MeltPro MTB/PZA showed 92.50% positive percent agreement and 97.78% negative percentage agreement, highlighting its diagnostic value. In conclusion, PZA resistance in multidrug-resistant tuberculosis (MDR-TB) is primarily due to *pncA* mutations. MeltPro MTB/PZA assay offers a reliable, rapid alternative for PZA resistance prediction, supporting timely treatment adaptations for improved TB patient care.

**IMPORTANCE:**

This study underscores the pressing need for reliable diagnostic methods to address high PZA resistance rates in TB cases, particularly in MDR-TB strains. By confirming that pncA mutations are the principal drivers of PZA resistance, we highlight the diagnostic potential of the MeltPro MTB/PZA assay as a rapid and effective alternative to conventional culture-based methods. Demonstrating sensitivity and specificity comparable to WGS and pDST, this assay offers a practical, accessible approach for timely PZA resistance prediction. It supports more tailored and effective MDR-TB treatment strategies, which are essential for optimizing patient care in both well-resourced and constrained settings.

## INTRODUCTION

Tuberculosis (TB), caused by *Mycobacterium tuberculosis* (MTB), remains a critical global health issue and stands as the second leading cause of mortality from a single infectious agent (1, 2). Despite significant advances in TB control throughout the 20th century, the emergence of multidrug-resistant TB (MDR-TB, resistant to at least isoniazid [INH] and rifampicin [RIF]) and pre-extensively drug-resistant TB (pre-XDR-TB, defined as MDR-TB with additional resistance to any fluoroquinolones) strains has introduced renewed challenges to global TB management and treatment (3, 4). Subsequently, the definition of pre-XDR-TB has been revised into the extensively drug-resistant TB (XDR-TB) by the World Health Organization (WHO), which is defined as infection with an MDR-TB strain that is also resistant to any fluoroquinolones and at least one additional group A drug (2). The widespread use of anti-tuberculosis drugs has contributed to the development of resistance within MTB populations, underscoring an urgent need for comprehensive public health strategies to address these drug-resistant TB variants (developed resistance to one or more anti-TB drugs) (5, 6).

Pyrazinamide (PZA) is a nicotinamide analog that belongs to the first line of intracellular bactericidal anti-tuberculosis drugs (7). Unlike other first-line anti-tuberculosis drugs, PZA is inactive against actively growing MTB at normal pH and is effective against slow-growing or “dormant” MTB (8, 9). PZA is usually used during the intensive phase of chemotherapy, which can shorten the treatment period from 9 to 12 months to 6 months. Because of PZA’s unique bactericidal effect, it is essential to detect PZA resistance in TB patients, especially in MDR-TB patients (10). However, PZA has a relatively high rate of resistance and is prevalent in rifampicin-resistant TB and MDR-TB strains, with PZA resistance rates ranging from 31% to 89% in different studies (9, 10).

Current understanding implicates mutations in drug-resistance genes as the primary genetic drivers of PZA resistance (11). The *pncA* gene, comprising 561 bp and encoding 187 amino acids, serves as the principal locus of drug resistance, with mutations therein either attenuating or abrogating the activity of the encoded pyrazinamidase/nicotinamidase, thereby compromising the conversion of PZA into pyrazinoic acid (POA) and contributing to MTB resistance (12). Nevertheless, the identification of PZA-resistant strains devoid of typical *pncA* mutations suggests the existence of alternative resistance mechanisms (13, 14). Investigations have highlighted *rpsA*, encoding the 30S ribosomal protein (S1), and *panD*, encoding aspartate decarboxylase, as potential alternative targets for POA action, although conclusive evidence supporting these assertions necessitates further experimental elucidation (15).

Phenotypic drug-susceptibility testing (pDST) remains the cornerstone method for evaluating drug resistance, particularly employing the MGIT 960 system (Becton Dickinson, Baltimore, MD, USA), regarded as the gold standard for PZA resistance detection (16). Notably, PZA activity is contingent upon specific acidic conditions (pH = 5.5) to impede MTB growth, with excessive inoculation precipitating pH elevation and enzymatic attenuation, potentially yielding false-resistant outcomes (17, 18). Despite the inherent limitations of traditional phenotypic assays, molecular techniques offer promising avenues for PZA resistance assessment (10). Molecular assays such as linear probe hybridization confer significant advantages in detecting drug-resistant mutations, including those associated with INH, RIF, and fluoroquinolones (FQs) (18, 19). Recently, Li et al. introduced a novel molecular assay, MeltPro MTB/PZA, exhibiting a detection sensitivity and specificity of 91.4% and 89.9%, respectively (9). Furthermore, whole-genome sequencing (WGS) affords comprehensive insights into drug resistance profiles across all anti-tuberculosis agents, enabling precise prediction of resistance even among strains with unreliable phenotypes (10).

In this study, we collected 125 clinical isolates to comprehensively evaluate the prevalence of PZA resistance and to elucidate the corresponding mutation profiles. The findings from pDST and WGS were utilized as benchmark controls, allowing us to analyze the clinical performance of the MeltPro MTB/PZA in accurately predicting PZA resistance, thereby facilitating timely and effective treatment strategies for patients.

## RESULTS

### Demographic characteristics and drug susceptibility profiles of clinical isolates

Among the 125 clinical isolates, patient ages ranged from 15 to 90 years, with a mean age of 43.34 years (SD = 14.26). Male patients were notably predominant, with 100 isolates (80.00%) derived from male patients and 25 isolates (20.00%) from female patients. In all cases, 87 (69.60%) had a history of prior treatment, while 38 (30.40%) were newly diagnosed. No statistically significant difference in PZA resistance rates was observed between the previously treated and newly diagnosed groups (*P* = 0.844).

Of the 125 MDR-TB isolates, 40.80% (51 of 125) strains were resistant only to INH and RIF, while 48.60% (67 of 125) were classified as pre-XDR-TB, and the remaining 5.10% (7 of 125) cases were identified as XDR-TB. The detailed characteristics of all isolates are summarized in Table 1. Among these strains, 74 (59.20%) were identified as PZA resistant, whereas 51 (40.80%) were deemed PZA susceptible. Notably, the prevalence of PZA-resistant strains was 77.61% in Pre-XDR-TB and 100% in XDR-TB, both significantly higher than the 29.41% observed in strains resistant only to INH and RIF.

**TABLE 1:** PZA resistance in different groups of TB patients

Characteristics	No. of isolates (*n* = 125)	No. of PZA-resistant isolates (*n* = 74)	No. of PZA-susceptible isolates (*n* = 51)	*P* value
Age (years)				
≤20	7	6	1	0.142
21–40	47	33	14	0.052
41–60	57	30	27	0.171
>60	14	5	9	0.058
Sex				
Male	100	55	45	0.056
Female	25	19	6	
Treatment history				
New case	38	22	16	0.844
Retreated	87	52	35	
Drug resistance to				
Strains resistant only to INH and RIF^*a*^	51	15	36	<0.001
Pre-XDR-TB^*b*^	67	52	15	<0.001
XDR-TB^*c*^	7	7	0	0.024

^*a*^ Strains resistant only to INH and RIF: strains resistant only to isoniazid and rifampicin.

^*b*^ Pre-XDR-TB: MDR-TB strains that were also resistant to any fluoroquinolones.

^*c*^ XDR-TB: MDR-TB strains that were also resistant to any fluoroquinolones and at least one other group A drug (bedaquinoline or linezolid).

### *PncA*, *rpsA*, and *panD* sequencing results

WGS analysis of 125 MDR-TB isolates revealed that 80 (64.00%) contained point mutations or insertion/deletion mutations within the *pncA* gene. Of these, 72 (90.00%) exhibited resistance to PZA, while 8 (10.00%) were PZA susceptible, as detailed in Table 2. Utilizing pDST as the reference standard, *pncA* sequencing alone demonstrated a sensitivity of 97.30% (95% CI, 89.69%–99.53%) and a specificity of 84.31% (95% CI, 70.86%–92.52%) with a kappa value of 0.831. Mutations in *rpsA* were identified in eight strains (6.4%), while no mutations were found in *panD*. It is noteworthy that six of the *rpsA* mutations occurred concomitantly with *pncA* mutations, while the remaining two strains (1.6%) had *rpsA* mutations alone. Individual sequencing of *rpsA* and *panD* for PZA resistance assessment showed limited sensitivity and lacked clinical significance but exhibited high specificity rates of 96.08% (95% CI, 90.75%–100.0%) and 100% (95% CI, 100%–100%), respectively. When *pncA* sequencing was combined with *rpsA* and *panD* mutations, the sensitivity remained at 97.30% (95% CI, 93.60%–100.0%), while specificity decreased to 80.39% (95% CI, 69.50%–91.29%), resulting in a kappa value of 0.796.

**TABLE 2:** Efficacy evaluation of sequencing for PZA resistance in MTB strains

Gene	PZA resistant (*n* = 74)		PZA susceptible (*n* = 51)		Sensitivity (%) (95% CI)	Specificity (%) (95% CI)	Accuracy (%) (95% CI)	Kappa value (95% CI)
	Substitutions Mutation and indel	No mutation and synonymous mutation	Substitutions Mutation and indel	No mutation and synonymous mutation				
*pncA*	72	2	8	43	97.30 (89.69–99.53)	84.31 (70.86–92.52)	92.00 (85.41–95.88)	0.831 (0.731–0.932)
*rpsA*	6	68	2	49	8.11 (1.89–14.33)	96.08 (90.75–100.0)	44.00 (35.30–52.70)	0.035 (−0.115 to 0.185)
*panD*	0	74	0	51	0 (0–0)	100 (100–100)	40.80 (42.18–49.42)	0 (−0.146 to 0.146)
*pncA*/*rpsA*/*panD*	72	2	10	41	97.30 (93.60–100)	80.39 (69.50–91.29)	90.40 (85.24–95.56)	0.796 (0.687–0.906)

### Characterization of *pncA* mutation

WGS analysis revealed mutations in the *pncA* and its promoter region in 80 (64%) isolates. The majority (92.50%) exhibited mutations within the coding region of *pncA*, while six (7.50%) displayed a singular −11A > G mutation in the promoter region (Table S1). Our investigation uncovered a diverse range of *pncA* mutation sites, comprising 53 distinct mutation types. Of these, 43 (81.13%) were attributed to single-nucleotide substitutions, while 10 (18.86%) were insertion/deletion events that may shift the amino acid reading frame (Table S1).

Despite the extensive diversity of *pncA* mutations, no predominant mutation type was identified. Notably frequent mutations included −11A > G (six, 7.50%) and V139A (five, 6.25%). Detailed information regarding mutation locations and frequencies within the *pncA* is presented in Table S1. Previous studies have highlighted three codon regions within the *pncA* coding sequence that are particularly enriched with mutations: 3–17, 61–85, and 132–142 (20). Analysis of mutation types within these regions revealed mutations in 12 strains (16.21%) in the 3–17 region, 7 strains (9.46%) in the 61–85 region, and 17 strains (22.97%) in the 132–142 region, collectively accounting for 48.65% of the entire *pncA* coding region. Most *pncA* mutations were characterized by single-nucleotide changes (79, 98.75%), with only one strain (1.25%) exhibiting multiple mutations (A46P, T76P, and V139G). Notably, no silent mutations were detected in this study, as detailed in Table S2.

### Clinical diagnostic performance of MeltPro MTB/PZA and WGS for PZA resistance

The aim of this study was to comprehensively evaluate the clinical efficacy of MeltPro MTB/PZA in predicting resistance to PZA. A total of 125 MDR-TB isolates underwent phenotypic and genotypic analyses, with pDST results obtained via the MGIT 960 method serving as the gold standard. As shown in Table 3, the MeltPro MTB/PZA method exhibited notable clinical sensitivity, specificity, and accuracy of 91.89% (95% CI, 85.67%–98.11%), 86.27% (95% CI, 76.83%–95.72%), and 89.60% (95% CI, 84.25%–94.95%), respectively, alongside a kappa value of 0.784. In comparison, WGS demonstrated clinical sensitivity, specificity, and accuracy of 97.30% (95% CI, 89.69%–99.53%), 84.31% (95% CI, 70.86%–92.52%), and 92.00% (95% CI, 85.41%–95.88%), with a kappa value of 0.831. Furthermore, the assay efficacy of the MeltPro MTB/PZA was compared with WGS, yielding a positive percent agreement (PPA) of 92.50% (95% CI, 86.73%–98.27%) and a negative percent agreement (NPA) of 97.78% (95% CI, 93.47%–100%), resulting in a kappa value of 0.881, indicating high diagnostic concordance (Table 4). These findings highlight the clinical utility of probe-based melting curve assays in detecting PZA resistance.

**TABLE 3:** Clinical performance of the pDST, MeltPro, and WGS assay

	pDST		Total	Sensitivity (%) (95% CI)	Specificity (%) (95% CI)	Accuracy (%) (95% CI)	Kappa value (95% CI)
	Resistant	Susceptible					
MeltPro							
Mutation	68	7	75	91.89 (85.67–98.11)	86.27 (76.83–95.72)	89.60 (84.25–94.95)	0.784 (0.673–0.895)
Wild type	6	44	50				
Total	74	51	125				
WGS							
Mutation	72	8	80	97.30 (89.69–99.53)	84.31 (70.86–92.52)	92.00 (85.41–95.88)	0.831 (0.731–0.932)
Wild type	2	43	45				
Total	74	51	125				

**TABLE 4:** Performance of the MeltPro assay for detection of resistance to PZA^*a*^

	WGS (*pncA*)			PPA (%) (95%CI)	NPA (%) (95%CI)	Accuracy (%) (95%CI)	Kappa value (95%CI)
MeltPro (*pncA*)	Mutation	Wild type	Total				
Mutation	74	1	75	92.50 (86.73–98.27)	97.78 (93.47–100.0)	94.40 (90.37–98.43)	0.881 (0.796–0.967)
Wild type	6	44	50				
Total	80	45	125				

^*a*^ NPA, negative percent agreement; PPA, positive percent agreement.

Additionally, there were 12 specimens with inconsistent results across pDST, WGS, and MeltPro MTB/PZA (Table S3). Among these, eight isolates were deemed susceptible by pDST but mutant according to the MeltPro MTB/PZA. All were found to have mutations via WGS. Conversely, four isolates exhibited drug resistance based on pDST yet were classified as wild type by MeltPro MTB/PZA. These samples also displayed mutations upon WGS analysis.

## DISCUSSION

PZA is a crucial intracellular bactericidal agent in anti-tuberculosis therapy, particularly noted for synergistic effects with other drugs against MDR-TB (9). While current phenotypic and genotypic methods effectively identify resistance to first-line drugs such as INH and RIF, the sensitivity testing for PZA remains inadequate, primarily due to false-positive results arising from acidic conditions and variations in inoculum size, which undermine diagnostic accuracy and reliability (18). Consequently, routine testing for PZA resistance faces significant laboratory limitations. In this study, we collected 125 MDR-TB isolates to assess the prevalence of PZA resistance in Wenzhou, Zhejiang Province, utilizing results from pDST and WGS as benchmarks to elucidate associated mutation characteristics. Additionally, we also introduced a rapid and straightforward MeltPro MTB/PZA detection method, which directly assesses mutations in the *pncA* gene and its promoter region following nucleic acid preparation. This method demonstrated high sensitivity and PPA in predicting PZA resistance compared to pDST and WGS, underscoring its potential as an effective screening tool in clinical practice.

Of the 125 isolates, 74 demonstrated resistance to PZA, resulting in a prevalence of 59.20%, significantly higher than national and regional averages (21, 22), indicating that Wenzhou is a hotspot for PZA resistance. Notably, 69.60% of these cases involved individuals who had received prior treatment, which may contribute to the elevated resistance rate. These findings highlight a concerning situation regarding PZA resistance in Zhejiang Province and underscore the urgent need for accurate PZA sensitivity testing to inform treatment strategies for MDR-TB.

Mutations in genes like *pncA*, *rpsA*, and *panD* are recognized as primary drivers of PZA resistance (12). In this study, WGS revealed a 64.00% mutation rate in *pncA*, notably lower than the national average of 80.00% (23), yet 97.30% of phenotyped PZA-resistant strains harbored *pncA* mutations, consistent with prior research (24). This study identified a total of 53 *pncA* mutation types across 80 isolates, encompassing 48 mutation sites, highlighting the stochastic and widespread nature of *pncA* mutations. The diversity of these mutations, particularly in key enzyme activity loci, poses diagnostic challenges, further complicated by the lack of prominent mutation hotspots (7, 25). Rare mutations in the upstream regulatory region of *pncA*, such as −11A > G, merit attention as they may influence PZA susceptibility (26–28). Interestingly, some PZA-resistant strains did not exhibit detectable *pncA* mutations, underscoring the importance of continued pDST to capture resistance variations (29). Additionally, several *pncA* mutations in PZA-sensitive strains did not affect susceptibility, illustrating the complexity of mutations. The role of *rpsA* and *panD* mutations in PZA resistance remains contradictory (16, 29). Our study reported a low *rpsA* mutation rate of 6.40%, with minimal impact on PZA resistance, consistent with previous findings (30). Furthermore, six *rpsA* nonsynonymous mutations, although associated with PZA resistance, co-occurred with *pncA* mutations, complicating efforts to isolate the role of *rpsA* mutations in resistance. *PanD* mutations were absent in our sample, potentially due to limitations in sample size. Thus, the relationship between *rpsA*/*panD* mutations and PZA resistance remains unclear, warranting further investigation.

Traditional culture-based pDST for PZA presents significant technical challenges and often yields unreliable results, underscoring the urgent need for novel molecular assays to detect PZA resistance and address the limitations of conventional methods. Our assessment of WGS for PZA resistance revealed high sensitivity and specificity with *pncA* sequencing alone, which outperformed individual *rpsA* and *panD* sequencing. Although sequencing all three genes together resulted in a slight reduction in specificity, *pncA* sequencing remains the preferred approach due to the technical difficulties associated with *rpsA* and its limited contribution to PZA resistance.

However, WGS is time-intensive, costly, and requires specialized technicians, making it impractical for rapid mutation detection to identify drug resistance in clinical settings, particularly in resource-limited environments. This limits the feasibility of WGS in primary laboratories (9). In contrast, MeltPro MTB/PZA presents a promising alternative to traditional culture-based tests, showing high sensitivity and specificity in assessing PZA resistance. Our study confirmed the clinical efficacy of the MeltPro MTB/PZA, demonstrating its ability to rapidly and accurately predict PZA resistance comparable to pDST and WGS. These findings support broader adoption of molecular assays to address the limitations of conventional methods.

In this study, WGS identified eight *pncA* mutations, while pDST showed sensitivity in these strains. According to the WHO’s second edition of the Tuberculosis Drug Resistance Mutations Catalogue (31), D129N and T47P are associated with PZA resistance, which may be due to changes in pH caused by inoculum size, leading to false-negative results in pDST (18). However, six mutations (G17C, F106C, T47I, V9A, T167I, and L35R) are not classified and may represent novel PZA mutations. Those unclassified *pncA* mutations may not be linked to PZA resistance, or their resistance effect may be insufficient to manifest as resistant in pDST, warranting further experimental validation. Four strains exhibited differences between MeltPro and WGS results (i.e., MeltPro wildtype vs WGS mutation). We attribute this discrepancy to the limitations of MeltPro in detecting certain types of mutations. Specifically, when the mutation is located near the ends of the probe, it may result in a minimal change in *T_m_* (less than 1.6°C). In these cases, MeltPro may classify the sample as wild type despite the presence of a mutation, leading to the observed discrepancies.

Despite the significant contributions of this study, several limitations exist. Firstly, eight *pncA* nonsynonymous mutations were detected in PZA-sensitive strains, potentially reflecting slight MIC increases that fall below the resistance threshold, hence categorized as PZA sensitive. Secondly, the reliability of pDST as the gold standard remains questionable, with potential false positives reducing the sensitivity of both the MeltPro MTB/PZA and WGS in clinical diagnostics. Lastly, in this study, which primarily included multidrug-resistant strains from a single region, the findings may lack broader applicability. Future studies with more diverse sample sets are warranted to strengthen result validity.

In summary, our study identified a high prevalence of PZA resistance among MDR-TB isolates from Wenzhou, Zhejiang Province, predominantly mediated by *pncA* mutations. The MeltPro MTB/PZA method demonstrated strong diagnostic performance as a rapid and precise molecular drug sensitivity assay, with results closely aligning with those from pDST and WGS. This method thus presents a valuable tool for the swift, early detection of PZA resistance, with WGS serving as a supplementary confirmatory approach.

## MATERIALS AND METHODS

### Clinical isolates

In total, 125 clinical isolates were randomly selected from preserved MDR-TB strains between 14 January 2014 and 30 August 2017 at Wenzhou Central Hospital, Zhejiang Province, China. This was a retrospective study, and the strains included were all stocked strains.

### Phenotypic drug susceptibility testing

Except for PZA, pDST was performed with the WHO-recommended proportion method (32). The concentrations of drugs in the Lowenstein-Jensen (L-J) medium were as follows: INH, 0.2 µg/mL; RIF, 40 µg/mL; ofloxacin, 2 µg/mL; levofloxacin, 2 µg/mL; and moxifloxacin, 1.0 µg/mL. The pDST for PZA was performed using a Bactec MGIT 960 PZA kit (Becton Dickinson). The critical concentration used was 100 mg/L for PZA (23). The standard strain of MTB H37Rv (ATCC 27294) supported by the National Tuberculosis Reference Laboratory was used as the sensitive control strain, while Bacillus Calmette–Guérin was used as the resistant control strain in each batch of tests.

### MeltPro MTB/PZA assay

DNA was extracted using an automated workflow (LabAid-824S; Zeesan Biotech, Xiamen, China) within 30 minutes of lysing the cultures with the provided buffer. We used the MeltPro MTB/PZA kit to predict MTB resistance to PZA, which was purchased from Xiamen Zeesan Biotechnology Co., Ltd. (Fujian, China), with catalog number 801021. This kit, based on multicolor melting curve analysis, detects mutations within the full coding region of the *pncA* gene (561 bp, PZA resistance-determining region) and the promoter region (−1 to −16) to identify MTB resistance to PZA. The reaction mixture was prepared according to the manufacturer’s instructions, and PCR was performed in the SLAN-96S thermocycler following the application protocols, with the results interpreted automatically by SLAN software (version 8.2) (Hongshi, Shanghai, China). The interpretation rules are as follows: PZA-resistant mutations are identified by comparing the differences in melting curve *T_m_* values (∆*T_m_*) between the tested sample and the wild type-positive control. If the *T_m_* values of the 36 melting peaks of the tested sample match those of the wild-type positive control (|∆*T_m_*| < 1.6°C), the result is considered susceptible. If any melting peak deviates from the wild-type positive control (|∆*T_m_*| ≥ 1.6°C), the result is considered resistant.

### Whole-genome sequencing

Strain DNA extraction, library construction, and sequencing followed methods detailed in our previous studies (33). Briefly, MTB colonies were scraped from the L-J medium, and genomic DNA was extracted using the QIAGEN Dneasy Blood & Tissue Kit per the manufacturer’s protocol. Sequencing libraries were prepared using the Nextera XT Sample Prep Kit (Illumina, San Diego, CA, USA) following standard protocol. All strains were sequenced on Illumina MiSeq, Illumina HiSeq, and BGISEQ500 platforms, achieving a minimum 100-fold coverage. Low-quality reads were filtered, and the remaining reads were aligned to the MTB H37Rv reference sequence (GenBank NC000962.3) using Bowtie2 (version 2.3.3.1) with default settings.

WGS data analysis adhered to established protocols. Single-nucleotide polymorphisms (SNPs) were identified using SAMtools (version 1.6) with a minimum depth of 10 reads, absence of strand bias, and a frequency threshold of ≥10%. All SNPs in PZA resistance-associated genes were statistically analyzed, with detailed criteria for resistance interpretation provided in reference 33 (Table S1).

### Statistical analysis

Clinical diagnostic performance, including sensitivity, specificity, PPA, and NPA, was calculated using two reference standards. For pDST as the reference, sensitivity and specificity of MeltPro MTB/PZA were calculated; for WGS as the reference, PPA and NPA of MeltPro MTB/PZA were calculated. The terms of PPA and NPA correspond to sensitivity and specificity, respectively, but are expressed differently, depending on the reference standard. Accuracy and kappa values were calculated for both comparisons. The formulas are as follows: sensitivity/PPA = true positive / (true positive + false negative); specificity/NPA = true negative / (true negative + false positive); accuracy = (true positive + false negative) / (true positive + false negative + true negative + false positive). Clinical evaluation results were analyzed using the OpenEpi online tool (https://www.openepi.com/) with 95% confidence intervals. The kappa value was calculated by SPSS (version 23.0; IBM, USA), with a kappa value of >0.75 indicating excellent consistency, a kappa value of <0.45 indicating poor consistency, and kappa = 0.45–0.75 indicating moderate consistency (34). Group differences were assessed with the chi-squared test, with significance set at *P* < 0.05.

## Data Availability

The raw sequence data reported in this paper have been deposited in the Genome Sequence Archive (Genomics, Proteomics & Bioinformatics 2021) at the National Genomics Data Center (Nucleic Acids Res 2022), China National Center for Bioinformation/Beijing Institute of Genomics, Chinese Academy of Sciences (GSA: CRA023489), and are publicly accessible at https://ngdc.cncb.ac.cn/gsa/browse/CRA023489.

## SUPPLEMENTAL MATERIAL

Supplemental material spectrum.02745-24-s0001.docx File S1; Tables S1 to S3.

ASM does not own the copyrights to Supplemental Material that may be linked to, or accessed through, an article. The authors have granted ASM a non-exclusive, world-wide license to publish the Supplemental Material files. Please contact the corresponding author directly for reuse.
