# Supplementary material for: Comprehensive evaluation of the MeltPro MTB/PZA assay for prediction of pyrazinamide resistance in multidrug-resistant tuberculosis
Source: Microbiol Spectr. 2025 May 22;13(7):e02745-24. doi: 10.1128/spectrum.02745-24 (PMC12210976; doi:10.1128/spectrum.02745-24)
Supplement: Supplemental material — File S1; Tables S1 to S3. [file spectrum.02745-24-s0001.docx]

**File S1 The Detection Principle of MeltPro MTB/PZA**

In the PCR system, probes labeled with fluorescent and quencher groups at both ends are added. During the PCR amplification process, single-stranded oligonucleotide sequences complementary to the probe sequence are amplified. After amplification, a melt curve analysis is performed, and the fluorescence changes are monitored in real-time. By calculating the negative derivative of fluorescence intensity with respect to temperature, the melt curve of the probe-target hybrid is obtained, allowing the determination of the melting temperature (*T_m_*) of the product, which provides mutation information of the sequence. If the target sequence perfectly matches the probe, the *T_m_* value of the probe-target hybrid will be the highest. However, if there is a mismatch between the probe and the sequence, such as a point mutation, insertion, or deletion, the *T_m_* value of the probe-target hybrid will be lower than that of the probe hybridized to a perfectly matched sequence. In short, the MeltPro MTB/PZA assay identifies mutations through changes in the *T_m_* value.

**Table S1. Mutant Profiles of MDR-TB Isolates Within *pncA***

|  | No. | Nucleotide Position | Nucleotide Change | Amino Position | Amino Acid Change | Mutation Type | No. of ioslates |
| --- | --- | --- | --- | --- | --- | --- | --- |
|  | 1 | -11 | A＞G | -11 | T to C | Non-synonymous | 6 |
| Spot mutation | 2 | 11 | T＞G | 4 | L to W | Non-synonymous | 4 |
|  | 3 | 14 | T＞C | 5 | I to T | Non-synonymous | 2 |
|  | 4 | 26 | T＞C | 9 | V to A | Non-synonymous | 2 |
|  | 5 | 29 | A＞G | 10 | Q to R | Non-synonymous | 1 |
|  | 6 | 42 | C＞G | 14 | C to W | Non-synonymous | 1 |
|  | 7 | 49 | G＞T | 17 | G to C | Non-synonymous | 2 |
|  | 8 | 56 | T＞G | 19 | L to R | Non-synonymous | 1 |
|  | 9 | 56 | T＞C | 19 | L to P | Non-synonymous | 1 |
|  | 10 | 83 | C＞A | 28 | A to D | Non-synonymous | 1 |
|  | 11 | 104 | T＞G | 35 | L to R | Non-synonymous | 1 |
|  | 12 | 123 | C＞G/A | 41 | Y to * | Nonsense | 3 |
|  | 13 | 137 | C＞T | 46 | A to V | Non-synonymous | 1 |
|  | 14 | 139 | A＞C | 47 | T to P | Non-synonymous | 1 |
|  | 15 | 139 | A＞G | 47 | T to A | Non-synonymous | 1 |
|  | 16 | 140 | C＞T | 47 | T to I | Non-synonymous | 1 |
|  | 17 | 146 | A＞G | 49 | D to G | Non-synonymous | 2 |
|  | 18 | 152 | A＞G | 51 | H to R | Non-synonymous | 1 |
|  | 19 | 161 | C＞G | 54 | P to R | Non-synonymous | 2 |
|  | 20 | 188 | A＞G | 63 | D to G | Non-synonymous | 1 |
|  | 21 | 199 | T＞C | 67 | S to P | Non-synonymous | 1 |
|  | 22 | 215 | G＞A | 72 | C to Y | Non-synonymous | 2 |
|  | 23 | 216 | C＞G | 72 | C to W | Non-synonymous | 2 |
|  | 24 | 254 | T＞G | 85 | L to R | Non-synonymous | 1 |
|  | 25 | 287 | A＞G | 96 | K to R | Non-synonymous | 1 |
|  | 26 | 287 | A＞C | 96 | K to T | Non-synonymous | 2 |
|  | 27 | 317 | T＞G | 106 | F to C | Non-synonymous | 1 |
|  | 28 | 357 | G＞T | 119 | W to C | Non-synonymous | 1 |
|  | 29 | 385 | G＞A | 129 | D to N | Non-synonymous | 1 |
|  | 30 | 391 | G＞T | 131 | V to F | Non-synonymous | 1 |
|  | 31 | 395 | G＞A | 132 | G to D | Non-synonymous | 1 |
|  | 32 | 401 | C＞A | 134 | A to D | Non-synonymous | 1 |
|  | 33 | 407 | A＞G | 136 | D to G | Non-synonymous | 1 |
|  | 34 | 416 | T＞C | 139 | V to A | Non-synonymous | 5 |
|  | 35 | 416 | T＞G | 139 | V to G | Non-synonymous | 2 |
|  | 36 | 415 | G＞C | 139 | V to L | Non-synonymous | 2 |
|  | 37 | 422 | A＞C | 141 | Q to P | Non-synonymous | 4 |
|  | 38 | 424 | A＞G | 142 | T to A | Non-synonymous | 1 |
|  | 39 | 500 | C＞T | 167 | T to I | Non-synonymous | 1 |
|  | 40 | 524 | T＞C | 175 | M to R | Non-synonymous | 1 |
|  | 41 | 524 | T＞G | 175 | M to T | Non-synonymous | 1 |
|  | 42 | 529 | A＞C | 177 | T to P | Non-synonymous | 1 |
| Deletion mutation |  |  |  |  |  |  |  |
|  | 43 | 7 | delG | 3 | Frameshift | Frameshift | 1 |
|  | 44 | 85 | delC | 29 | Frameshift | Frameshift | 1 |
|  | 45 | 510_*135 | 510_*135del |  | Frameshift | Frameshift | 1 |
|  | 46 | 528 | delC | 176 | Frameshift | Frameshift | 1 |
| Insertion mutation |  |  |  |  |  |  |  |
|  | 47 | 51 | insC | 18 | Frameshift | Frameshift | 1 |
|  | 48 | 192 | insA | 65 | Frameshift | Frameshift | 1 |
|  | 49 | 281 | insA | 94 | Frameshift | Frameshift | 1 |
|  | 50 | 393 | dupC | 131 | Frameshift | Frameshift | 1 |
|  | 51 | 399 | dupT | 133 | Frameshift | Frameshift | 1 |
|  | 52 | 419 | dupGC | 140 | Frameshift | Frameshift | 1 |
| Multiple-mutation |  |  |  |  |  |  |  |
|  | 53 | 381，226，416 | DupGG，A>C，T>G | 46，76，139 | A46P, T76P, V139G | Non-synonymous | 1 |

Note: 510_*135del indicates deletion of base 510 to base 135 downstream of the stop codon in the DNA sequence of the *pncA* gene.

**Table S2. *PncA* Mutants in Different Groups of TB Patients**

|  | No. of PZA Susceptible | No. of PZA Resistant | Total |
| --- | --- | --- | --- |
| No mutation | 43 | 2 | 45 |
| Single mutation | 8 | 71 | 79 |
| Silent mutation | 0 | 0 | 0 |
| Multiple-mutation | 0 | 1 | 1 |
| Total | 51 | 74 | 125 |

**Table S3. Discordance between pDST, WGS and Melt Pro MTB/PZA**

| **Sample.ID** | **Phenotypic DST** | **WGS** | **Melt Pro MTB/PZA** |
| --- | --- | --- | --- |
| pDST-S vs WGS-MT vs Melt Pro-MT（N=8） |  |  |  |
| 16-2715 | S | *pncA*_G17C | R |
| 16-440 | S | *pncA_*F106C | R |
| 15-333 | S | *pncA_*T47I | R |
| 15-2221 | S | *pncA_*L35R | R |
| B105 | S | *pncA*_D129N | R |
| C794 | S | *pncA*_T167I | R |
| 14-84 | S | *pncA_*V9A | R |
| D755 | S | *pncA*_T47P | R |
| pDST-R vs WGS-MT vsMelt Pro-WT（N=4） |  |  |  |
| 14-80 | R | *pncA*_A46P, *pncA*_T76P, *pncA*_V139G | S |
| 15-1948 | R | *pncA*_85G_del | S |
| 16-815 | R | *pncA*_Q141P | S |
| B20 | R | *pncA*_192T_in | S |

Note: S indicates susceptible, R indicates resistant, MT indicates mutant type, WT indicates wild type.
